# Aluminium release and fluid warming: provocational setting and devices at risk

**DOI:** 10.1186/s12871-021-01378-7

**Published:** 2021-05-27

**Authors:** Thorsten Perl, N. Kunze-Szikszay, A. Bräuer, M. Quintel, T. Roy, K. Kerpen, U. Telgheder

**Affiliations:** 1 Department of General, Visceral and Pediatric Surgery, University Medical Center Goettingen, Robert-Koch-Str. 40, D- 37075 Goettingen, Germany; 2 Department of Anaesthesiology, University Medical Center Goettingen, Goettingen, Germany; 3 Faculty of Chemistry, Departments of Instrumental Analytical Chemistry, University Duisburg-Essen, Essen, Germany

**Keywords:** Hypothermia, Prevention, Warming techniques, Fluid warming

## Abstract

**Background:**

Fluid warming, recommended for fluid rates of > 500 ml h^-1^, is an integral part of patient temperature management strategies. Fluid warming devices using an uncoated aluminium containing heating element have been reported to liberate aluminium resulting in critical aluminium concentrations in heated fluids. We investigated saline solution (0.9%), artificially spiked with organic acids to determine the influence of fluid composition on aluminium release using the uncoated enFlow® device. Additionally, the Level1® as a high volume fluid warming device and the ThermoSens® device were investigated with artificial spiked fluid at high risk for aluminum release and a clinically used crystalloid solution.

**Results:**

Saline solution spiked with lactate more than acetate, especially at a non neutral pH, led to high aluminium release. Next to the enFlow® device, aluminium release was observed for the Level1® device, but not for the coated ThermoSens®-device.

**Conclusion:**

Uncoated aluminium containing fluid warming devices lead to potentially toxic levels of aluminium in heated fluids, especially in fluids with non-neutral pH containing organic acids and their salts like balanced electrolyte solutions.

**Supplementary Information:**

The online version contains supplementary material available at 10.1186/s12871-021-01378-7.

## Summary

Aluminium release by anodized und uncoated fluid warming devices may lead to concerning aluminium concentration in intravenous fluids. The chemical background of this problem is addressed in this investigation with six fluids and two different fluid warming devices.

## Introduction

Fluid warming is next to active body surface warming and prewarming an integral part of patient temperature management and is recommended for fluid amounts of more than 500 ml or fluid rates of > 500 ml h^− 1^ [1, 2]. For fluid warmers using anodized aluminium as a heating element, a concerning aluminium release to fluids passing the heating element was demonstrated [3, 4]. After this observation, the Medicines and Healthcare products Regulatory Agency (MHRA) reacted immediately with an information about potentially higher than expected aluminium levels by using the enFlow® fluid warming device (VyAire Medical Inc., Mettawa, IL, USA), and after confirmation of these results with Medical Device Alerts (MDA). Finally, these observations [3, 4] led to withdrawal of the device by manufacturer.

However, fluid warming devices as medical products undergo defined test in approval procedures by notified bodies, including tests for inadvertent leaching of substances like aluminum. Although high aluminium releases have only been reported for anodized fluid warming devices that dispense on coating, for coated devices (like a parylene coating) an aluminium release would be possible. Disruptions in the thin parylene layer might develop under influence of heating in normal application or due to failures in production an aluminium release is thinkable.

Test protocols use saline solutions as test fluid. Meanwhile, excessive aluminium release with an uncoated, anodized heating element was observed mainly for a balanced electrolyte solution and much lesser for a saline solution [4]. A further investigation to characterize the aluminium release by the enFlow® device described corresponding high amounts of aluminium with lactated solutions [3]. In contrast, the amount of aluminium release using human albumin solution, fresh frozen plasma and resuspended, expired red cells was much lesser [3]. The exact mechanism or chemical condition facilitating the release of aluminium from anodized heating elements remains unclear. Identification of the fluid composition with both, clinically relevant compounds and capacity of aluminum release will allow to refine testing protocols for approval of fluid warming devices. For clinicians the knowledge about the risk of inadvertent aluminum exposition for patients is important and both have to be taken into account, the type of device (coated or uncoated) and the applied fluid composition is important to avoid unnecessary aluminum exposition.

The aim of this study was to shed some light to the chemical conditions facilitating aluminium release. To assess the composition of fluids leading to maximum aluminium release the enFlow® device, known for critical aluminium release, was tested applying saline solution artificial spiked with several compounds. In a second step we investigated the amount of aluminium release by two other previously not tested fluid warming devices with an aluminium containing heating element applying the previously identified risk setting with the identified artificial solution and additionally a clinical used standard balanced electrolyte solution.

## Methods

### Investigated solutions

To identify the most reactive substances of a balanced electrolyte solution we created several solutions (Table 1). Furthermore, a clinical used balanced electrolyte solution (Sterofundin® ISO 1/1 E ISO, B. Braun Melsungen AG, Melsungen, Germany) was used. Electrolyte concentrations of this balanced fluid are as followed: sodium 145.0 mmol l^− 1^; potassium 4.0 mmol l^− 1^; magnesium 1.0 mmol l^− 1^; calcium 2.5 mmol l^− 1^; chloride 127.0 mmol l^− 1^; acetate 24.0 mmol l^− 1^; malate 5.,0 mmol l^− 1^.

**Table 1:** List of used fluids. Saline 0.9 % (B.Braun Melsungen AG, Melsungen, Germany) was used pure ① or spiked with organic acid-salt (column 2) and additionally pH-modified (column 3). All chemicals were purchased from Fisher-Scientific (Schwerte, Germany) [1] or Sigma-Aldrich (Munich, Germany) [2]

Number	Saline 0.9 % spiked with	pH modification	CAS
①	pure	-	
②	50 mmol Sodium acetate	-	CAS 127-09-3 [1]
③	50 mmol sodium DL-lactate	-	CAS 867-56-1 [2]
④	50 mmol sodium DL-lactate	hydrochloric acid (pH 4)	CAS 867-56-1 [2]
⑤	50 mmol sodium DL-lactate	Sodium hydroxide (pH 9)	CAS 867-56-1 [2]
⑥	pure	hydrochloric acid (pH 4)	

### Measurements

Prepared fluids were pumped with a peristaltic pump (Infusomat® fms, B. Braun Melsungen AG, Melsungen, Germany) at a flow rate of 4 ml min^− 1^ through the fluid warming device. All fluids were tested with an uncoated device known for critical aluminium release [3, 4] (enFlow®, Vital Signs, Inc., aGE Healthcare Company, Totowa New Jersey, USA). Baseline measurements were performed from samples before the fluids passed into the heating device. Instantly after baseline measurements, fluids were pumped through the warming units for 60 min. Samples of the heated infusion fluids were collected after 30 and 60 min at the distal end of the infusion line. All samples were analysed with a graphite furnace atomic absorption spectrometry (GFAAS) (GFAAS AA6800/6650 Shimadzu Corporation, Japan). Each measuring sequence was performed 6 times with a new infusion warming disposable. All results are presented as median (IQR [range]).

The limit of detection (LOD) and the limit of quantification (LOQ) for the determination of aluminium were calculated according to standard DIN 32,645 (German Institute for Standardisation), allowing for dilution [5]. The LOD is the lowest quantity of a substance that can be distinguished from the absence of that substance with 99 % and was 4 µg l^− 1^ (0.15 µmmol l^− 1^). The LOQ is the limit at which the difference between two distinct values can be reasonably discerned and was 14 µg l^− 1^ (0.52 µmol l^− 1^).

### Tests of two fluid warming devices

In a second step two fluid warmers with a potential risk of aluminium release due to aluminium containing heating element were tested:

- The level 1® (Level 1® H-1025 and DI-300 disposable, Smith medical, Minneapolis, USA). This device is a high-volume fluid warmer designed to warm fluids with flow rates up to 1400 ml min^− 1^ and uses an uncoated aluminium tube as a heat exchanger.
- The ThermoSens® (Barkey GmbH & Co. KG, Leopoldshöhe, Germany). This device is designed for fluid warming with flow rates of up to 150 ml min^− 1^ and uses a coated aluminum heating chamber as a heat exchanger.

According to the results of the first experiment, we choose the saline solution (0.9 %) spiked with 50 mmol sodium DL-lactate titrated to a pH of 9 and a commercial, balanced electrolyte solution (Sterofundin®ISO 1/1 E ISO, B. Braun Melsungen AG, Melsungen, Germany). Flow rate (4 ml min^− 1^) and sample time (baseline, 0 min., 30 and 60 min.) were same like in the first experiment.

## Results

**Fig. 1:**
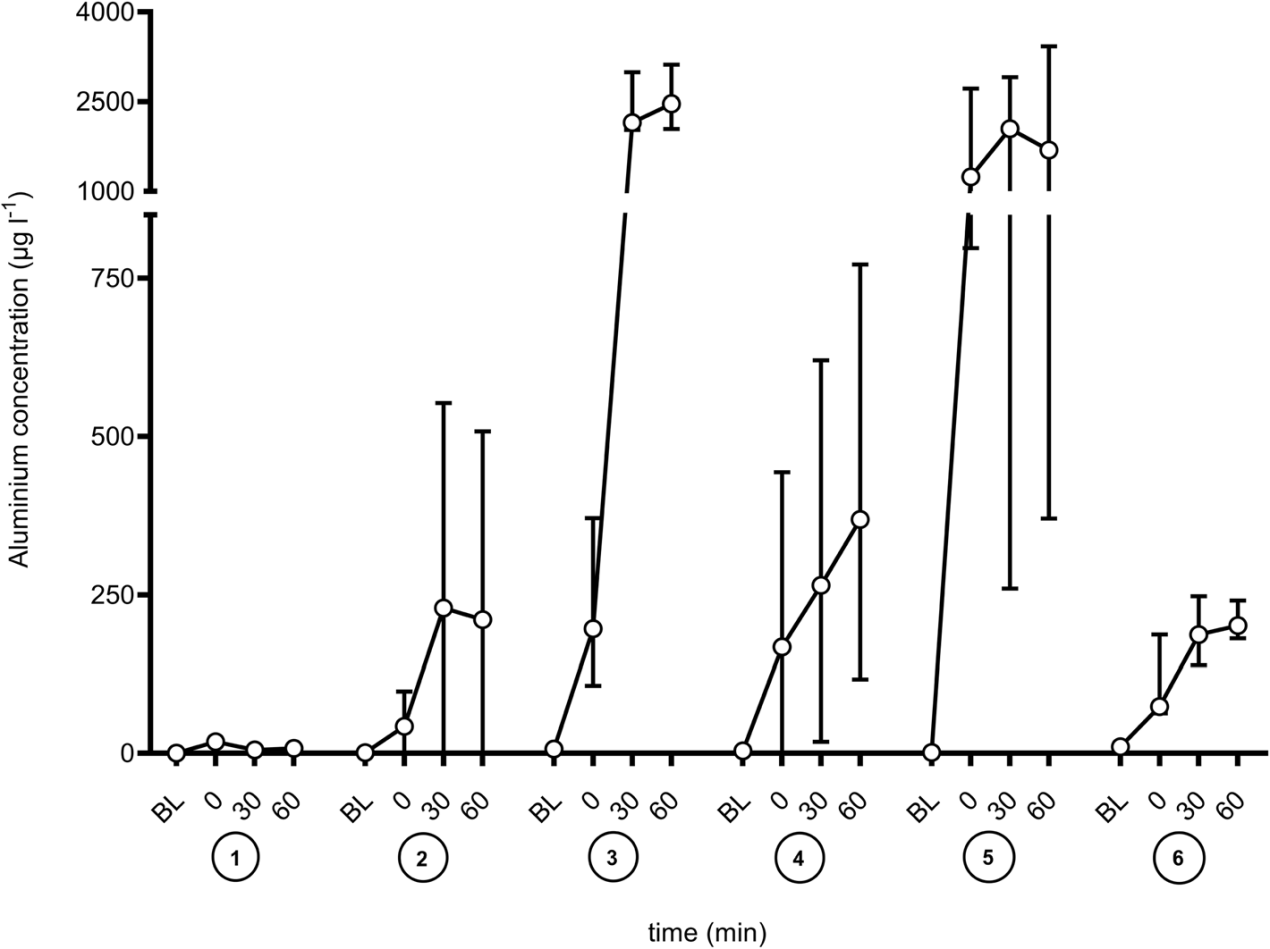
Mean (SD) aluminium concentration over the time (BL – Baseline; 0 to 60 min; each six measurements) for the enFlow® (Vital Signs, Inc., aGE Healthcare Company, Totowa New Jersey, USA) with different fluids as follows: ① saline 0.9 % ② saline 0.9 % with 50 mmol acetate ③ saline 0.9 % with 50 mmol lactate ④ saline 0.9 % with 50 mmol lactate at pH 4.0 ⑤ saline 0.9 % with 50 mmol lactate at pH 9 ⑥ saline 0.9 % at pH 9. Each six measurements

Results of the enFlow® device with saline solutions are displayed in Fig. 1. If saline was used as a fluid, the aluminium concentration was 7.6 (6-8.2 [5.5–8.8]) µg l^− 1^ (0.28 (0.22–0.3 [0.2–0.33]) µmol l^− 1^) after 60 min. Highest aluminium concentrations were observed when saline was spiked with 50 mmol lactate at a pH 9 induced by sodium hydroxide. The aluminium concentrations were determined to 2462.4 (2109.6-2954.3 [1921.1-3379.8]) µg l^− 1^ (91.36 (78.27–109.6 [71.27-125.39]) µmmol l^− 1^) and 1692 (433.1-3152.4 [282.8-3938.6]) µg l^− 1^(62.77 (16.07-116.95 [10.49-146.12]) µmol l^− 1^). In lactate spiked saline at a pH of 4 induced by hydrochloric acid the aluminium concentration was determined to 369.4 (157.1-624.7 [29.6-1134.7]) µg l^− 1^ (13.7 (5.83–23.18 [1.1–42.1]) µmol l^− 1^) after 60 min. Aluminium concentration in saline spiked with 50 mmol acetate was determined to 210.6 (0-450.2 [0-537]) µg l^− 1^ (7.81 (0-16.7 [0-19.92]) µmol l^− 1^) after 60 min (Fig. 1).

Saline spiked with lactate in a not acidified solution was identified to be most corrosive and therefore used for the tests with the two additional tested fluid warming devices.

**Fig. 2:**
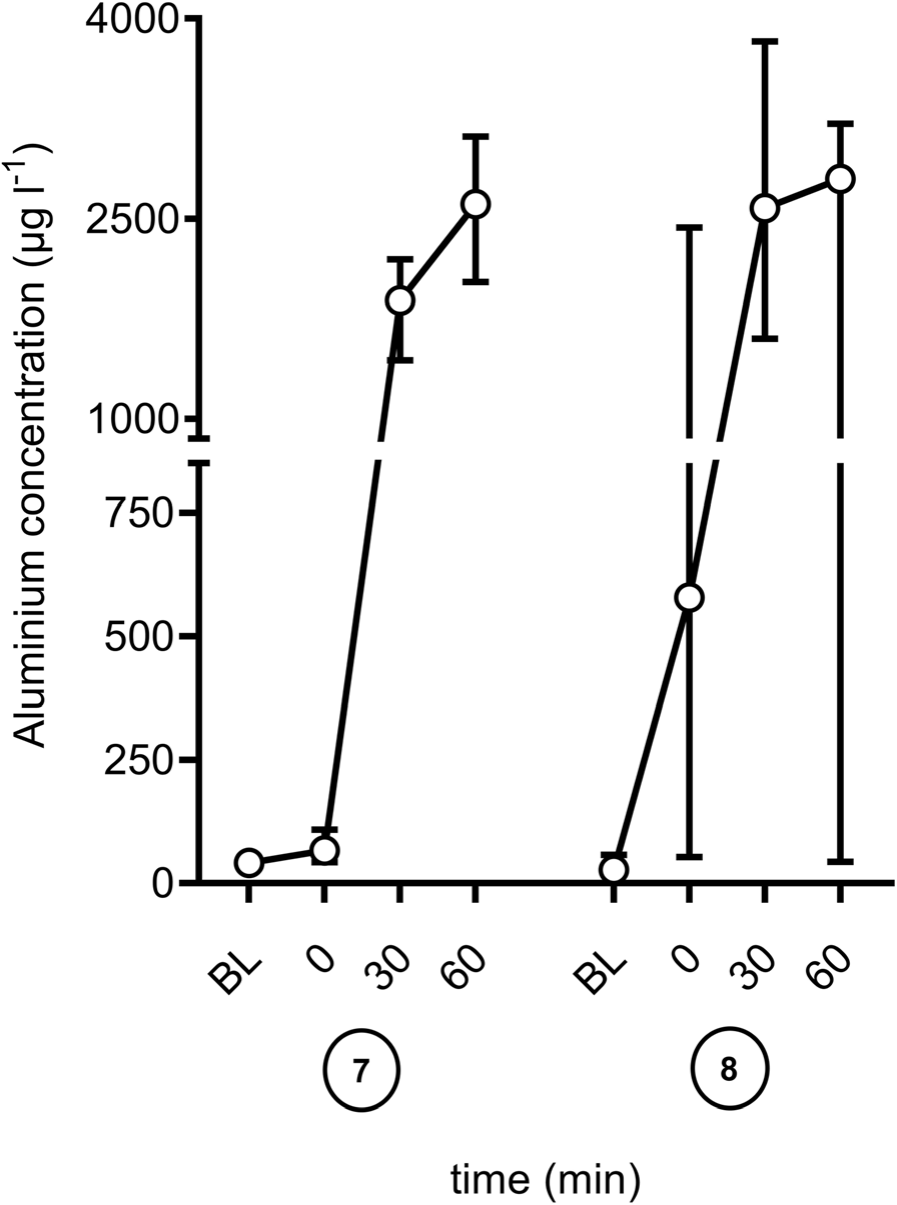
Mean (SD) aluminium concentration over the time (BL – Baseline; 0 to 60 min; each six measurements) for the Level 1® (H-1025 and DI-300 disposable, Smith medical, Minneapolis, USA) with different fluids as follows: ⑦ saline 0.9 % with 50 mmol lactate ⑧ saline 0.9 % with 50 mmol lactate at pH 9

Aluminium concentration in saline spiked with 50 mmol lactate after 60 min perfusion with a high flow fluid warmer (Level 1® H-1025 and DI-300 disposable, Smith medical, Minneapolis, USA) was determined to 2609.3 (2135.6–3043 [1891.8-3172.2]) µg l^− 1^ (96.81 (97.23–112.9 [70.19-117.69]) µmol l^− 1^) and titrated to a pH of 9 with sodium hydroxide 2798.8 (691.7-3050.7 [6.9-3645.7]) µg l^− 1^ (103.84 (25.66-113.18 [0.26-135.26]) µmol l^− 1^) (Fig. 2). The same setting of fluids warmed with a coated low flow device (ThermoSens®, Barkey GmbH & Co. KG, Leopoldshöhe, Germany) led to not quantifiable aluminium concentrations.

**Fig. 3:**
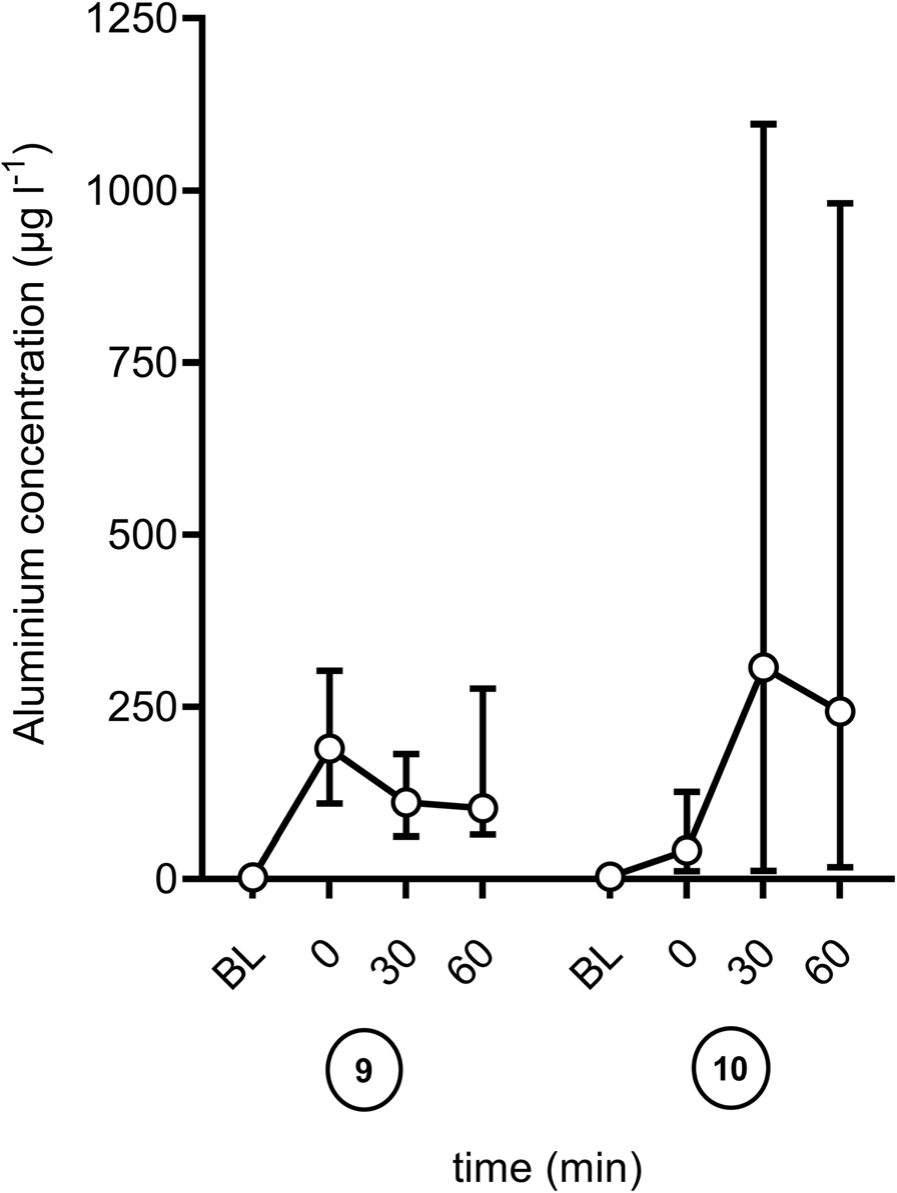
Mean (SD) aluminium concentration over the time (BL – Baseline; 0 to 60 min; each six measurements) with balanced electrolyte solution Sterofundin 1/1 E ISO: ⑨ EnFlow® (Vital Signs, Inc., aGE Healthcare Company, Totowa New Jersey, USA) ⑩ Level 1® (H-1025 and DI-300 disposable, Smith medical, Minneapolis, USA)

The analysis of the standard balanced fluid (Sterofundin 1/1 ISO) after flowing 60 min through the three tested devices revealed to aluminium concentrations of 243 (65.2–400 [10.9-2608.6]) µg l^− 1^ 9.02 (2.42–14.84 [0.4-96.78]) µmol l^− 1^) for the Level1® and 102.6 (80.6-209.6 [35.8-371.8]) µg l^− 1^ (3.81 (2.99–7.78 [1.33–13.79]) µmol l^− 1^) for the enFlow® device (Fig. 3). There was no aluminium detectable using the ThermoSens® (Barkey GmbH & Co. KG, Leopoldshöhe, Germany) device.

## Discussion

The main result of our study is that lactate with non-neutral pH is most effective in aluminium release with uncoated heating devices. It was confirmed that sodium chloride solution itself has only very limited capacity of aluminum release. However, aluminium concentrations determined by analysing acetate buffered sodium solutions or commercial balanced electrolyte solutions, which are common in clinical routine, are reasonable.

Aluminium is one of the most studied toxic metals and associated with many diseases [6] like encephalopathies such as Alzheimers [7], impaired neurologic development of (preterm) infants [8] and osteomalacia [9]. Physiologic aluminium concentration in whole blood is 1–2 µg l^− 1^ (0.04–0.07 µmol l^− 1^), with a transferrin binding of > 90 % [10]. Although soluble aluminium in plasma underlies fast renal clearance, but therefore is dependent on renal function. A deposition of up to 20 % is possible [11].

One possible safety threshold is the recommended threshold for parenteral nutrition as this threshold reflects an intravenous administration. For preterm infants with need of parenteral nutrition a repeated intravenous aluminum supply of > 4–5 µg kg^− 1^ d^− 1^ is a risk for encephalopathy, impaired neurologic development and osteomalacia [8, 9]. The American Society for Clinical Nutrition (ASCN) and the American Society for Parenteral and Enteral Nutrition (ASPEN) therefore recommended a threshold of 25 µg l^− 1^ [12] (0.93 µmol l^− 1^) for aluminium content of parenteral nutrition which is also noted by the United States FDA [13] .

A different approach is to apply the oral minimum risk level for aluminium, derived by the Agency for Toxic Substances and Disease Registry to be at 1 mg.kg^− 1^ d^− 1^ [14, 15]. Aluminium shows a poor bioavailability with only 0.1 % resorption after oral administration [16]. If the calculated threshold with a correction factor of 1000 based on the bioavailability is applied, the proposed calculations lead to a tolerable parenteral minimal risk level of not more than 1 µg kg^− 1^ day^− 1^ or 70 µg kg^− 1^ day^− 1^ for a 70 kg adult. However, the nonrecurring use of a fluid warmer is different to long term use of parenteral nutrition or a daily oral dose and therefore the limits cannot be transferred directly to the occasional use of fluid warming. A third way of estimating the maximum safe aluminium exposure is to apply the threshold for aluminium salt in vaccines of 850 µg dose^− 1^ [17]. To date there are no regulations for maximal tolerable aluminium concentrations of iv fluids available.

Even if these values are inconsistent, it can be assumed that any kind of aluminium infusion is inadvertent and a dosage of more than 1000 µg for adults is unsafe. Regarding the results of this investigation not only the concentration of aluminium in the warmed fluid alone is relevant. For the estimation of safety, the concentration must be multiplied with the administrated volume. Fluid warming is indicated and recommended fluid rates of more than 500 ml h-1. For bleeding patients e.g., after major trauma high volume fluid demands of more than 5 l.h-1 are reasonable. In consequence the observed concentrations of aluminium liberated by uncoated fluid warming devices as enFlow® and Level1® are both not safe and bear a potential risk of aluminium intoxication and maybe long-term effects like enzephalopathia [18, 19]. Our study confirms the observation of aluminum release by the enFlow® device [3, 4] and the Level1 [20]. Highest described aluminium concentrations for the uncoated enFlow® device yielded aluminium concentrations of up to 6794 µg l^− 1^ (252.06 µmol l^− 1^) in mean using a balanced, lactate buffered electrolyte solution at a flow rate of 4 ml min^− 1^ [4]. A second publication described mean values of 4386.6 µg l^− 1^ (162.5 µmol l^− 1^) using lactated saline solution and 6027.9 µg l^− 1^ (223.2 µmol l^− 1^) for Plasma-Lyte 148 [3] using the uncoated enFlow® device with applied flow rates of 2 ml min^− 1^. An investigation using the high flow device Level 1® using Lactated Ringer’s solution at a higher flow rate of 30 ml min^− 1^ described an aluminium concentration of 278.5 µg l^− 1^ (10.3 µmol l^− 1^) [20]. Higher observed aluminum concentrations in this study compared to the investigation of Cabrera et al. [20] may be explained by using a high flow disposable (Level1® DI-300) in this investigation rather than the moderate flow disposable (Level1® DI-100) [20]. The difference between these disposables is a higher efficacy for the DI-300, presumably by enlarged surface of the heat exchanging element. A limitation of the study design is, that a flow rate of 4 ml min^− 1^ does not reflect the range in which high volume fluid warming devices are typically used. However, even is concentration might be significant lower under influence of increased flow rate, the effect on total amount of leached aluminium would be less. The influence of flow rate on resulting aluminium concentration (and amount of aluminium) an aspect for future investigations.

In the previous study [4] the increase of temperature was associated with an increase in aluminum release by an uncoated device. Temperature is influential and heat may theoretically enhance both chemical reactions and dissolution rates. It was also demonstrated previously that the flow rate influences aluminum concentration. Higher flow rates are accompanied with lower aluminum concentrations, but of course the amount of aluminum infused will be less affected. The results from the current investigation describe a strong effect of pH on aluminum release mediated by organic acids (lactate, acetate or malate) and their salts. Since alumina is amphoteric, alumina can be dissolved in either acidic or alkaline solution. The reaction of Al-containing material depends on the total concentration of aluminium, solution pH, and the presence of complex-forming ligands. Depending on the solution pH range different polymeric species exist. At a pH of 3–5, positively charged Al species are predominantly present (e.g.Al_3_(OH)_4_^5+^, Al_13_(OH)_32_^7+^) [21]. The mole fraction of these species is proportional to the concentration of Al (III). At a pH < 4, mainly Al^3+^ is present and Al(OH)^2+^ exists in the pH range between 4 and 6, while Al(OH)_4_^−^ is predominant at pH > 8. At a pH of 9, aluminium hydroxide species is formed and this is coordinated to the lactic acid or the corresponding salt [22]. At a high lactate concentration, the aluminium complex is also formed to a greater extent. But unlike acetic acid, lactic acid forms a stable complex with aluminium. This finding is in accordance with Figs. 1 and 2. At the same time, this effect may also explain the results of an investigation of the enFlow® device using blood products [3] reporting rather low levels of aluminium in expired packed red blood cells because these blood products do not only contain high levels of lactate but also have a low pH. The association of non-neutral pH and predominant Al(OH)_4_^−^Ions is relevant for clinical settings, as buffered electrolyte solutions with pH > 7 are in daily routine widespread used.

The strength of a bench investigation is the standardized examination of aluminium release. However, a problem of any bench investigation is that no resulting plasma concentrations of aluminium can be reported. Assessment of any patient outcomes such as cognitive dysfunction induced by the infused aluminium is impossible. However, the exposition of patients resulting from uncoated fluid warming devices using aluminium as heating element can be estimated and bear a potential risk of toxic levels.

In conclusion, uncoated fluid warming devices using aluminium as heating element bear the risk of aluminium release in a potential toxic amount. Organic acids and their salts like lactate and acetate are compounds provoking the reaction, especially under non-neutral conditions. Test protocols for leaching of aluminium from fluid warming devices should apply lactate spiked saline better than saline solution. In clinical context the use of balanced electrolyte fluids with uncoated fluid warming devices using aluminium heating elements is associated with a risk of inadvertent aluminium exposition of patients.

## Supplementary Information

**Additional file 1**

## Data Availability

All data generated or analysed during this study are included in this published article and its supplementary information files. [raw data.xlsx]

## References

[1] NICE: Hypothermia: prevention and management in adults having surgery. Clinical guideline [CG65]**.** In: NICE Clinical Guideline. https://www.nice.org.uk/guidance/cg65: NICE Clinical Guideline; 2016.

[2] Torossian A, Brauer A, Hocker J, Bein B, Wulf H, Horn EP (2015). Preventing inadvertent perioperative hypothermia. Dtsch Arztebl Int.

[3] Taylor MH, Choi D, Fitzpatrick SM, Gunn KN (2019). Characterisation of aluminium release by the enFlow(R) fluid-warming system in crystalloids and blood products. Anaesthesia.

[4] Perl T, Kunze-Szikszay N, Brauer A, Quintel M, Rohrig AL, Kerpen K, Telgheder U (2019). Aluminium release by coated and uncoated fluid-warming devices. Anaesthesia.

[5] (DIN) DIfN: Chemical analysis - Decision limit, detection limit and determination limit under repeatability conditions - terms methods, evaluation. In: DIN 32645:vol. 32645; 2008.

[6] Fulgenzi A, Vietti D, Ferrero ME: Aluminium involvement in neurotoxicity. BioMed Res Int. 2014, 2014:758323. 10.1155/2014/758323 PMC4160616 25243176

[7] Kawahara M, Kato-Negishi M (2011). Link between Aluminum and the Pathogenesis of Alzheimer’s Disease: The Integration of the Aluminum and Amyloid Cascade Hypotheses. Int J Alzheimers Dis.

[8] Bishop NJ, Morley R, Day JP, Lucas A (1997). Aluminum neurotoxicity in preterm infants receiving intravenous-feeding solutions. N Engl J Med.

[9] Klein GL (1995). Aluminum in parenteral solutions revisited–again. Am J Clin Nutr.

[10] Krewski D, Yokel RA, Nieboer E, Borchelt D, Cohen J, Harry J, Kacew S, Lindsay J, Mahfouz AM, Rondeau V (2007). Human health risk assessment for aluminium, aluminium oxide, and aluminium hydroxide. J Toxicol Environ Health B Crit Rev.

[11] Talbot RJ, Newton D, Priest ND, Austin JG, Day JP (1995). Inter-subject variability in the metabolism of aluminium following intravenous injection as citrate. Hum Exp Toxicol.

[12] Charney PJ (2004). American Society for P, Enteral Nutrition Aluminum Task F: A.s.p.e.N. Statement on aluminum in parenteral nutrition solutions. Nutr Clin Pract.

[13] US FDA (US Food and Drug Administration). Aluminum in large and small volume parenterals used in total parenteral nutrition: proposed rule. Federal Register. 1998;63:176–85. 10176836

[14] EFSA. Safety of aluminium from dietary intake—scientific opinion of the panel on food additives, flavourings, processing aids and food contact materials (AFC). EFSA J. 2008;(7):754. 10.2903/j.efsa.2008.754 PMC10193631 37213837

[15] Ingerman L, Jones DG, Keith S, Rosemond ZA: Toxicological profile for aluminum. 2008.

[16] Willhite CC, Karyakina NA, Yokel RA, Yenugadhati N, Wisniewski TM, Arnold IM, Momoli F, Krewski D (2014). Systematic review of potential health risks posed by pharmaceutical, occupational and consumer exposures to metallic and nanoscale aluminum, aluminum oxides, aluminum hydroxide and its soluble salts. Crit Rev Toxicol.

[17] Baylor NW, Egan W, Richman P (2002). Aluminum salts in vaccines–US perspective. Vaccine.

[18] Exley C (2020). Aluminium-based fluid warmers are not proven to be safe. Anaesthesia.

[19] Perl T, Kunze-Szikszay N, Bräuer A, Roy T (2020). Quantified aluminium levels released into blood and fluids using the Level 1 Fast Flow Fluid Warmer. Anaesthesia.

[20] Cabrera JA, Borton LK, Barrett G (2020). Quantified aluminium levels released into blood and fluids using the Level 1 Fast Flow Fluid Warmer. Anaesthesia.

[21] Nguyen TTN, Lee MS (2019). Speciation of alumina in aqueous solution and its interaction with silicate ion. Geosyst Eng.

[22] Sen AB, Kapoor SN. Coordination Compounds of Aluminium with Lactic Acid. J für Praktische Chemie. 1963;22(5–6):314–8.

